# Quality of care in obstetric services in rural South India: evidence from two studies with gap of ten years

**DOI:** 10.1186/1753-6561-6-S1-O2

**Published:** 2012-01-16

**Authors:** Asha Kilaru, Saraswathy Ganapathy, Baneen Karachiwala

**Affiliations:** 1 Belaku Trust, Bangalore, India

## Introduction

With a high maternal mortality ratio (of 212 per 100000 live births), provision of quality maternal healthcare services has remained a challenge in India. In 2005, government of India launched the National Rural Health Mission (NRHM) to improve healthcare services and health status of population in rural India with a focus on maternal and child health.

In this paper we compared findings from two studies conducted with a gap of nearly ten years to understand changes in maternal healthcare services before and after the NRHM. While the study population belonged to rural Karnataka, many of the issues identified represent the rural population across the country.

## Methods

We compared the findings from two studies conducted with a gap of ten years in a rural block of Ramanagara district in Karnataka. These studies explored various aspects of the pregnancy related healthcare delivery by healthcare services as well as the way care was experienced by women and their families. Table 1 provides salient features of these studies.

**Table 1:** Study design

	Study 1	Study 2
Duration	1996-1998	2007-2009
Methods	Prospective cohort study with use of mixed methods.	Prospective cohort study with use of mixed methods.
Sample	11 villages selected randomly. Recruitment of all pregnant women (520) between 1996-98 from these villages. Followed to 3 months postpartum.	39 villages randomly selected across 13 primary health centres. Subsequently 41 villages purposively (located adjacent to villages selected earlier) selected to meet enrolment target. All women who planned to deliver within study area and were in the third trimester of pregnancy were enrolled (642). 608 (94.7%) women completed the study.
Data collection	Five questionnaires (two antenatal, one immediately post-delivery and one about three months post-partum) were administered.	Two questionnaires (one antenatal in the third trimester, one about one month after delivery) were administered.

## Results

We found that in the first study, 30% of women delivered at other than their planned place of delivery for reasons excluding direct referrals from healthcare services. This level remained similar (33%) in the second study indicating little change in birth preparedness and emergency planning.

Overall, proportion of deliveries happening at healthcare institutions increased from 35% to over 80%. Skilled birth attendance by Auxiliary Nurse Midwife (ANM) in case of deliveries happening at home reduced from 34% to 17% in spite of the lower incidence of home births. The number and appropriate timings of antenatal care visits improved including the content of care package (blood pressure examination, iron and folic acids supplements) across the two time periods.

However there were gaps in certain aspects of antenatal care such as urine test, advise from healthcare services regarding risk signs in pregnancy and timings for postpartum visit. We found from second study that patient-provider interactions were generally poor and that providers were unlikely to be aware of the socio-cultural beliefs in community. The first study showed that such socio-cultural beliefs in community influence women’s’ and families' responses to illness.

We found that the median cost for households for normal deliveries ranged between INR 1000 (USD 21.5) to INR 4000 (USD 85.9) across primary health centres, block (*taluk*) and tertiary level government hospitals. Median cost for caesarean section at tertiary care government hospitals was INR 8000 (USD 171.8).

Furthermore, we found that certain measure quality of care such as of postpartum counselling, early postpartum check-ups, length of stay, appropriate use of uterotonics, and equity in service provision across caste groups needed improvement.

## Discussion

By comparing the findings of two studies over a decade, we show that there have been some improvements in pregnancy related healthcare services. However many challenges remain. We suggest that use of checklists by healthcare providers on specific components of recommended care during antenatal check-ups, delivery and postnatal care (for example, birth planning during antenatal visits, postpartum and neonatal advice given before discharge) is needed.

There is need to increase the length of stay for women in institution to at least 6 hours post delivery for all women with uncomplicated normal deliveries and with healthy babies and longer for women with complications and/or low birth weight babies. Specific attention to questions that women and their families may have regarding healthcare as well as allowing women to have companion of their choice present during delivery would help improve care experienced by women.

There is need to improve round the clock presence of doctors and healthcare workers at primary health centres because many women had to seek other facilities as they found primary health centres closed. We found that the cost of care in government services was higher than the cash assistance available through *janani suraksha yojana* (maternity protection scheme) to families living below poverty line.

